# The genetic feature and virulence determinant of highly virulent community-associated MRSA ST338-SCCmec Vb in China

**DOI:** 10.1080/22221751.2021.1914516

**Published:** 2021-06-06

**Authors:** Ye Jin, Wangxiao Zhou, Zhidong Yin, Shuntian Zhang, Yunbo Chen, Ping Shen, Jinru Ji, Weiwei Chen, Beiwen Zheng, Yonghong Xiao

**Affiliations:** a State Key Laboratory for Diagnosis and Treatment of Infectious Diseases, National Clinical Research Center for Infectious Diseases, Collaborative Innovation Center for Diagnosis and Treatment of Infectious Diseases, The First Affiliated Hospital, Zhejiang University School of Medicine, Hangzhou, People’s Republic of China; b Department of Pathology, The Second Affiliated Hospital of Zhejiang University College of Medicine, Hangzhou, People’s Republic of China; c Department of Laboratory Medicine, The First Affiliated Hospital, College of Medicine, Zhejiang University, Hangzhou, People’s Republic of China

**Keywords:** CA-MRSA, bloodstream infection, Panton–Valentine leucocidin, core-virulence factors, ST338

## Abstract

ST59 is the predominant pathotype of community-associated methicillin-resistant *Staphylococcus aureus* (CA-MRSA) in China. As a variant of ST59, there is relatively little known about the detailed information of ST338. To address this issue, here, we described thirteen ST338 CA-MRSA strains isolated from severe bloodstream infection cases, and focused on their epidemiology, genetic features and virulence potential. Phylogenetic analysis showed the earliest isolated strain of this study is likely a predecessor of recent ST338 lineage (after year of 2014). Furthermore, the phylogenetic reconstruction and time estimation suggested that ST338 evolved from ST59 in 1991. Notably, the carrying patten of virulence factors of all ST338 strains were similar, and the genomic islands νSaα, νSaγ and SaPI and the core virulence factors like *hla* and *psm* were detected in ST338 isolates. However, all ST338 isolates lacked some adhesion factors such as *clfA*, *clfB*, *eap*, *cna* and *icaD*. Additionally, among these ST338 strains, one PVL-negative ST338 isolate was detected. Experiment on mice nose and human alveolar epithelial cell showed that the nasal colonization ability of ST338 was weaker than that of CA-MRSA MW2. In a mouse bloodstream infection model and skin infection model, PVL+ and PVL− strains had the similar virulence, which was dependent on upregulation of toxin genes rather than the presence of mobile genetic elements such as ΦSa2 carrying PVL. Our findings provide important insight into the epidemiology and pathogenicity of the novel and highly virulent ST338-SCC*mec* Vb clone.

## Introduction

*Staphylococcus aureus* is a common opportunistic pathogen that usually colonizes human skin and nasal mucosa and causes a variety of infections ranging from mild skin lesions to severe diseases such as endocarditis, osteomyelitis, pneumonia, and even life-threatening septic shock [1–3].

*S. aureus* can acquire resistance in various ways, which has led to the emergence of community-associated methicillin-resistant *S. aureus* (CA-MRSA), a major cause of infections in hospitals worldwide [4], and the most common cause of skin and soft tissue infections (SSTIs) in the United States [5–7]. Unlike healthcare-associated (HA)-MRSA, which is mostly isolated from older patients with underlying health problems and risk factors [8], CA-MRSA tends to infect healthy younger individuals and is more virulent, potentially causing severe pneumonia and fatal sepsis [9]. Some CA-MRSA clones have a high rate of dissemination within the general population.

CA-MRSA harbours type IV or V *staphylococcal* chromosomal cassette *mec* (SCC*mec*) elements that are smaller than the type II or III SCC*mec* elements of HA-MRSA [10]. Unlike HA-MRSA, CA-MRSA strains are sensitive to many non-β-lactam antibiotics and carry Panton–Valentine leukocidin (PVL) genes [11]. CA-MRSA strains were once limited to populations outside health care settings, which was the basis for their differentiation from HA-MRSA. However, this distinction has been obscured in recent years and the prevalence of CA-MRSA strains has increased; thus, the epidemiology of MRSA has changed with the emergence of CA-MRSA strains in healthcare settings. The epidemiologic success of CA-MRSA is attributed to a combination of low fitness cost of antibiotic resistance and high virulence [12–14]. However, most studies on CA-MRSA in China have focused on the ST59 clone, and there is little information on other clones.

ST59, the predominant CA-MRSA clone in China, has 2 variants: a PVL+ Taiwan clone (ST59-SCC*mec* V) and PVL− Asia-Pacific clone (ST59-SCC*mec* IV) [15–17]. Studies conducted in Taiwan have reported that ST59-SCC*mec* V has a greater capacity to lyse neutrophils and causes more severe infections than ST59-SCC*mec* IV, although the latter more efficiently colonizes nasal mucosa [18, 19]. ST338 is a variant of ST59, with a single-nucleotide mutation in the housekeeping gene *gmk*. The virulent ST59-SCC*mec* Vb clone recently emerged in Taiwan and mainland China [20, 21], and its virulence determinants have been investigated [22], but detailed information on ST338-SCC*mec* Vb is lacking.

The Blood Bacterial Resistance Investigation Collaborative System (BRICS) is a national surveillance programme that collects pathogens isolated from patients with bloodstream infection to monitor epidemics of bacteria in China [23–25]. From January 2014 to December 2019, we isolated 927 MRSA strains from various provinces and cities in China; 13 of these were ST338-SCCmec Vb (SA01–SA13) classified as CA-MRSA according to the Centers for Disease Control and Prevention definition and caused severe bloodstream infections. In this study, we report 13 cases of severe septicaemia caused by SA01–SA13 and describe the epidemiology, genetic features, and virulence of the isolates. Among these CA-MRSA isolates, one PVL-negative ST338 isolate was detected. Thus, to identify the key elements in the evolution of virulence in CA-MRSA, we subsequently analysed the virulence factors in CA-MRSA ST338-SCC*mec* Vb.

## Materials and methods

### Bacterial strains and CA-MRSA definition

ST338 isolates were obtained from BRICS. CA-MRSA was defined as a strain isolated within 48 h from outpatients or patients admitted to the hospital who had no history of inpatient contact with nursing homes, shelters, or other health institutions within the previous 12 months; and had no indwelling catheter or artificial medical device.

### Antimicrobial susceptibility testing

Antimicrobial susceptibility was assessed using the VITEK 2/VITEK 2 XL system (BioMérieux, Marcy l’Etoile, France). The minimum inhibitory concentrations (MICs) of 17 antimicrobial agents including ciprofloxacin, nitrofurantoin, levofloxacin, penicillin G, sulfamethoxazole and trimethoprim, teicoplanin, linezolid, tigecycline, quinupristin/dalfopristin, gentamicin, oxacillin, rifampin, tetracycline, vancomycin, clindamycin, moxifloxacin, and erythromycin were determined.

### Whole-genome sequencing (WGS) and genomic analysis

Genomic DNA of ST338 and ST59 isolates was extracted using the Ezup Column Bacteria Genomic DNA Purification Kit (Sangon Biotech, Shanghai, China) and sequenced using the HiSeq X 10-PE150 platform (Illumina, San Diego, CA, USA). The quality of raw reads was verified using FastQC 0.11.7 software [26] and the reads were trimmed using Trimmomatic v0.34 (Phred quality score >20) [27]. Clean reads were assembled using SPAdes v3.13 [28] with default settings. The assembly was filtered and contigs larger than 200 bp were retained. Multilocus sequence typing of *S. aureus* was performed by searching 7 housekeeping genes against the National Center for Biotechnology Information (NCBI) PubMLST database (https://github.com/tseemann/mlst). SCC*mec* typing was performed using SCCmecFinder (https://cge.cbs.dtu.dk/services/SCCmecFinder/). The sequences of 30 ST338 and 30 ST59 MRSA-SCC*mec* Vb strains from China and surrounding geographic regions were downloaded from GenBank for comparisons.

### Phylogenetic analysis

We used Snippy v4.6.0 (https://github.com/tseemann/snippy) to identify single-nucleotide polymorphisms (SNPs) in the ST338 and ST59 strains, with M013 (GenBank accession no. CP003166) – a PVL+ CA-MRSA ST59 SCC*mec* Vb clone isolated in Taiwan [29] – as a reference. Recombined regions were detected using Gubbins v2.4.1 [30], and the output from Gubbins was used as the input for BactDating v1.0 [31] to perform phylogenetic dating based on a Bayesian approach. The Markov chain Monte Carlo chain lengths were run for 100 million cycles to convergence; the effective sample size of the inferred parameters α, μ, and σ was >200. A minimum spanning tree (MST) based on SNP data from the 13 ST338 SCC*mec* Vb isolates in this study was constructed using CiteSpace [32].

### Identification of virulence genes and detection of *S. aureus* pathogenicity islands (SaPIs)

Sequences of *S. aureus* MW2 (ST1; GenBank accession no. BA000033), USA300 (ST8; GenBank assembly accession GCA_000013465.1), rh10 (ST59-SCC*mec* Vb from mainland China), and M013 (ST59-SCC*mec* Vb from Taiwan; GenBank accession no. CP003166) were used for comparative analyses of virulence. Genome sequences were annotated using the RAST server (https://rast.nmpdr.org/rast.cgi). Virulence factors were detected using the Virulence Factors Database analyser (http://www.mgc.ac.cn/VFs/). A genomic island map was generated using the genoplotR package of R software [32].

### Mouse nasal colonization model

Animal experiments were carried out in accordance with the guidelines for the Care and Use of Laboratory Animals of the Chinese Association for Laboratory Animal Sciences. The mouse nasal colonization model was established using 6-week-old female BALB/C mice purchased from the Animal Laboratory Center of the First Affiliated Hospital of Zhejiang University School of Medicine. A drop of phosphate-buffered saline (PBS; 20 μl) containing 1×10^8^ *S. aureus* cells was introduced into the nasal passage of the mice (*n*=5 per *S. aureus* strain). After 3 days, the mice were sacrificed and the nose was excised and homogenized. Total *S. aureus* counts were determined by plating 200 µl of diluted nose tissue cell suspension on tryptic soy agar (TSA).

### Adhesion of *S. aureus* to human alveolar epithelial cells

The adhesion assay was performed as previously described [33]. Briefly, strains were grown for 6 h at 37°C and then washed 3 times with sterile PBS. A549 human alveolar epithelial cells and bacterial cells were coincubated at 37°C and 5% CO_2_ in a 6-well plate. After 3 h, dissociated bacterial cells were washed and 0.1% deoxysodium cholate solution was used to lyse the A549 cells.

### Bacteraemia caused by MRSA in a mouse sepsis model

Female BALB/C mice (6 weeks old) were used to establish a sepsis model. Each mouse was housed in the laboratory for 10 days prior to the experiment. *S. aureus* strains were grown for 9 h and cells were harvested by centrifugation and washed 3 times with PBS; 1×10^8^ cells were resuspended in 50 μl sterile PBS and injected into mice (*n*=10 per strain) via the caudal vein. Negative control mice were injected with 50 μl sterile PBS. The physical condition of inoculated mice was monitored and recorded daily. Mice were sacrificed as soon as they were unable to eat or drink. When the first mouse infected with ST338 died, all remaining mice in the group were sacrificed. At 7 days post infection, all surviving mice were sacrificed. The lungs were dissected, fixed in 10% formalin, and embedded in paraffin. Sections were cut and stained with haematoxylin and eosin. The left kidney was removed and homogenized in 1 ml PBS, and 200 μl of homogenized tissue was diluted and plated on TSA for *S. aureus* cell counts.

### Quantitative reverse-transcription (qRT)-PCR

RNA was extracted from *S. aureus* strains using the RNeasy Purification Plus Kit (Qiagen, Hilden, Germany). Purified RNA was reverse transcribed to cDNA using SYBR Green Premix (Takara Bio, Otsu, Japan). Expression of *hla*, *psmα*, *agrA*, and *RNAIII* genes in ST338 was detected by qRT-PCR and quantified with the 2^−ΔΔCt^ method. USA300 was used as a reference strain (relative expression=1) because of its high endogenous expression of these genes [34, 35].

### Red blood cell (RBC) lysis assay

Haemolytic capacity was evaluated as previously described [36]. Briefly, *S. aureus* strains were grown for 9 h and centrifuged. The supernatant was incubated with rabbit RBCs for 3 h at 37°C. Purified water and rabbit RBCs resuspended in NaCl solution served as positive and negative controls, respectively.

### Cytotoxicity assay

Neutrophils were extracted from the blood of healthy human volunteers using the Human Neutrophil Isolation Solution Kit (Sangon Biotech) and stored in cell-preserving solution. *S. aureus* strains were grown overnight at 37°C. After 1:200 dilution in PBS, 200 μl of the culture and neutrophils were coincubated in a 96-well microplate for 3 h at 37°C. Neutrophils incubated in 0.1% Triton-X100, TS broth, and cell-preserving solution served as positive, negative, and blank controls, respectively.

### Statistical analysis

Assays were performed three times. The q test was used to evaluate differences in skin lesions between mice, and the Wilcoxon rank-sum test was used to compare colony-forming unit (CFU) counts. Survival rates of mice infected with *S. aureus* were analysed with the Kaplan–Meier method. qRT-PCR data were analysed with the unpaired *t* test. Results of the haemolysis test and cytotoxicity assay were analysed with the chi-squared test. Data analyses were performed with Prism v7.0 software (GraphPad, San Diego, CA, USA). *P*<0.05 was considered significant.

## Results

### Brief case reports

**Figure 1.**
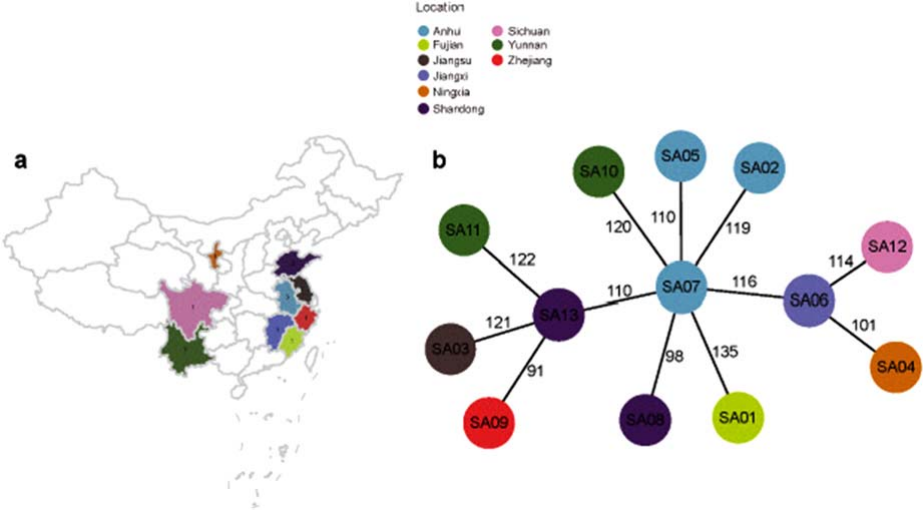
Relationships among ST338 isolates in this study. (a). Geographic distribution of clinical cases of ST338 infection in China from 2014 to 2019. Different colours represent areas where the strains were isolated. (b). MST of ST338 isolates. The genome sequences of 13 ST338 isolates were aligned. Each circle represents a ST338 strain, and each colour represents a different geographic region. Numbers on the connecting lines indicate the number of single nucleotide polymorphisms between two strains.

Of the 927 MRSA isolated from blood samples in China over a 6-year period, 13 ST338-SCC*mec* Vb isolates (ST338-SCCmec Vb; SA01–SA13) were obtained from severe CA-MRSA infection cases at different hospitals [Figure 1(a)]. The mean age of patients was 32 years old. Nine cases were primary SSTIs; 7 patients ultimately died. The clinical information of these patients is presented in Table 1.

**Table 1.** Clinical reports of ST338 community-associated methicillin-resistant *Staphylococcus aureus* cases.

Strains/case	Case information	Detailed information						
	Year	Location	Age	Sex	Primary disease	Treatment	Outcome	
SA01/Case 1	2016	Fujian	32	Male	SSTI	Vancomycin	Cured	A previously healthy 32-year-old male presented with a skin abscess on his back. His body temperature was 39.1°C; leukocyte count was 11.76×10^9^/l, with 79.9% neutrophils. ST338 was detected in the lesion and blood. He was treated with vancomycin and discharged.
SA02/Case 2	2016	Anhui	51	Male	SSTI	Vancomycin	Cured	A previously healthy 51-year-old male presented with SSTI on his right lower extremity resulting from fracture of the tibia and fibula after a fall 10 days before hospitalization. His body temperature was 38.6°C. The lesions on the patient’s right lower limb and tibia were necrotic, with a large amount of purulent secretion. MRSA ST338 was isolated from the lesions and blood. Under epidural anaesthesia, the ruptured skin was repaired with a prefabricated skin flap. After the operation, the patient received anti-infective therapy and other supportive treatments.
SA03/Case 3	2017	Jiangsu	58	Female	SSTI	Vancomycin	Cured	A 58-year-old woman with systemic lupus erythematosus presented with skin and soft tissue lesions on the back of her left hand. Her body temperature was 38.2°C; leukocyte count was 16.28×10^9^/l; and CRP level was 119.00 mg/l. After 2 days of hospitalization, the patient experienced pericardial effusion and dyspnoea. MRSA ST338 was isolated from the skin lesions and blood. The patient underwent abscess drainage and was treated with vancomycin before being discharged.
SA04/Case 4	2017	Ningxia	49	Female	IE	Vancomycin	Death	An otherwise healthy 49-year-old woman presented with chest pain and petechial rash that had persisted for 2 weeks and 5 days, respectively. She had arrhythmia and her heart rate was 85 bpm; apical diastolic murmurs were detected. Body temperature was 39.6°C; leukocyte count was 14.77×10^9^/l, with 93.20% neutrophils. An echocardiogram revealed severe mitral valve stenosis and vegetation and tricuspid regurgitation. MRSA ST338 was detected in blood cultures. The patient was treated with vancomycin, but died of heart failure 4 days after admission.
SA05/Case 5	2018	Anhui	2 months	Male	Pneumonia	Vancomycin	Death	An otherwise healthy 2-month-old baby presented with fever, productive cough, and shortness of breath that had persisted for 3 days. Breathing rate was 32 breaths/min. Body temperature was 38.5°C; leukocyte count was 7.22×10^9^/l, with 58.60% neutrophils. A chest radiograph revealed pneumonia. MRSA ST338 was detected in the sputum and blood. The patient was treated with vancomycin; however, sepsis and brain abscess occurred, and he died 2 weeks after hospitalization.
SA06/Case 6	2018	Jiangxi	24	Female	SSTI	Vancomycin	Cured	A previously healthy 24-year-old woman presented with fever (38.7°C). Four days prior, multiple purulent skin and soft tissue lesions had developed on her back. Leukocyte count was 38.87×10^9^/l, with 87.80% neutrophils. MRSA ST338 was detected in the skin lesions and blood. The patient was treated with a parenteral dose of vancomycin. The lesions were drained and treated with mupirocin ointment. The patient was discharged 10 days after admission.
SA07/Case 7	2014	Anhui	42	Male	Pneumonia	Vancomycin	Death	A 42-year-old man presented with cough and oliguria that had persisted for 4 days. His body temperature was 39.2°C; leukocyte count was 20.46×10^9^/l, with 83.90% neutrophils. CRP level was 212.59 mg/l. Two days after hospitalization, the patient experienced kidney failure. MRSA ST338 was detected in his blood. The patient was treated with vancomycin but died 1 week later.
SA08/Case 8	2015	Shandong	8	Male	SSTI	Vancomycin	Death	A previously healthy 8-year-old boy presented with a skin abscess on his right leg. His body temperature was 39.8°C; leukocyte count was 15.83×10^9^/l, with 85.8% neutrophils. The diagnosis was acute suppurative osteomyelitis. MRSA ST338 was detected in the lesion and blood. The patient was treated with vancomycin but died from sepsis 12 days after hospitalization.
SA09/Case 9	2016	Zhejiang	25	Male	SSTI	Vancomycin	Death	A 25-year-old healthy man presented with fever (38.5°C) and coma. Before hospitalization, a skin lesion was observed on his forehead. Leukocyte count was 11.56×10^9^/l and CRP level was 270.07 mg/l. Anti-infective treatment (1 g vancomycin every 12 h) was initiated before aetiology was established, but the patient died 5 h after admission. Two days later, the presence of MRSA ST338 in blood samples was confirmed.
SA10/Case 10	2019	Yunnan	18	Male	SSTI	Vancomycin	Cured	A previously healthy 18-year-old male presented with a skin abscess on his finger. His body temperature was 38.9°C; leukocyte count was 15.76×10^9^/l, with 86.9% neutrophils. ST338 was detected in the lesion and blood. He was cured after 7 days of treatment with vancomycin.
SA11/Case 11	2019	Yunnan	29	Male	SSTI	Vancomycin	Cured	A 29-year-old healthy woman presented with a skin abscess on the back of her hand. Physical examination revealed a leukocyte count of 12.38×10^9^/l and CRP level of 180.63 mg/l. She was cured after treatment with vancomycin.
SA12/Case 12	2019	Sichuan	5	Female	Pneumonia	Vancomycin	Death	A 5-year-old girl presented with fever, productive cough, and shortness of breath. An isolate was obtained from the patient’s blood and identified as MRSA ST338. The patient was treated with vancomycin but died 1 week later.
SA13/Case 13	2019	Shandong	70	Male	SSTI	Vancomycin	Death	An otherwise healthy 70-year-old man was admitted to the hospital with high fever. His right leg had a festering lesion that was determined to be an SSTI. Three days after admission, the patient showed signs of septic shock. MRSA ST338 was isolated from a blood sample. The patient died 2 weeks after admission.

CRP, C-reactive protein; IE, infective endocarditis; MRSA, methicillin-resistant *Staphylococcus aureus*; SSTI, skin and soft tissue infection.

### Characteristics of ST338 isolates

The 13 MRSA isolates were identified as ST338 SCC*mec* Vb (5C2&5) CA-MRSA strains (Table 2). All isolates were resistant to erythromycin, clindamycin, and oxacillin and had a methicillin MIC of 4 μg/ml, indicating very low methicillin resistance. The isolates were susceptible to trimethoprim, vancomycin, rifampin, levofloxacin, moxifloxacin, gentamicin, teicoplanin, amikacin, tigecycline, daptomycin, and linezolid.

**Table 2.** Genotypes and susceptibilities to 17 antibiotics in thirteen clinical MRSA isolates of ST338 in China.

Strains/Case	Antimicrobial agents																
	Year	ERY	CLI	OXA	SXT	TCY	VAN	RIF	CIP	LVX	MFX	GEN	TEC	AMK	TGC	DAP	LNZ
SA01/Case1	2016	R	R	R	S	R	S	S	R	S	S	S	S	S	S	S	S
SA02/Case2	2016	R	R	R	S	S	S	S	S	S	S	S	S	S	S	S	S
SA03/Case3	2017	R	R	R	S	S	S	S	S	S	S	S	S	S	S	S	S
SA04/Case4	2017	R	R	R	S	R	S	S	S	S	S	S	S	S	S	S	S
SA05/Case5	2018	R	R	R	S	R	S	S	S	S	S	S	S	S	S	S	S
SA06/Case6	2018	R	R	R	S	S	S	S	S	S	S	S	S	S	S	S	S
SA07/Case7	2014	R	R	R	S	S	S	S	S	S	S	S	S	S	S	S	S
SA08/Case8	2015	R	R	R	S	R	S	S	S	S	S	S	S	S	S	S	S
SA09/Case9	2016	R	R	R	S	S	S	S	R	S	S	S	S	S	S	S	S
SA10/Case10	2019	R	R	R	S	S	S	S	S	S	S	S	S	S	S	S	S
SA11/Case11	2019	R	R	R	S	S	S	S	S	S	S	S	S	S	S	S	S
SA12/Case12	2019	R	R	R	S	S	S	S	S	S	S	S	S	S	S	S	S
SA13/Case13	2019	R	R	R	S	S	S	S	S	S	S	S	S	S	S	S	S

ERY: erythromycin; CLI: clindamycin; OXA: oxacillin; SXT: trimethoprim-sulfamethoxazole; TCY: tetracycline; VAN: vancomycin; RIF: Rifampin; CIP: ciprofloxacin; LVX: levofloxacin; MFX: moxifloxacin; GEN: gentamicin; TEC: teicoplanin; AMK: amikacin; TGC: Tigecycline; DAP: daptomycin; LNZ: linezolid.

### Relationships among ST338 isolates in this study

To determine whether the CA-MRSA ST338 strains causing severe bloodstream infection are an epidemiologic threat, we screened 927 MRSA strains isolated from 17 provinces in China from 2014 to 2019. With the exception of Ningxia Province, all strains emerged in geographically close provinces [Figure 1(a)], suggesting clonal transmission of ST338-SCC*mec* Vb. The MST revealed that all ST338 isolates were closely related (<150 SNPs). Strain SA07 [middle circle in Figure 1(b)] was the earliest isolate identified in Anhui Province in 2014, while other strains were isolated after 2014, implying that SA07 is the ancestor of the other ST338-SCC*mec* Vb clones and has the potential to evolve by adapting to different environments through the acquisition of novel mutations.

### Phylogenetic construction of ST338 SCC*mec* Vb clonotype MRSA and time estimation

**Figure 2.**
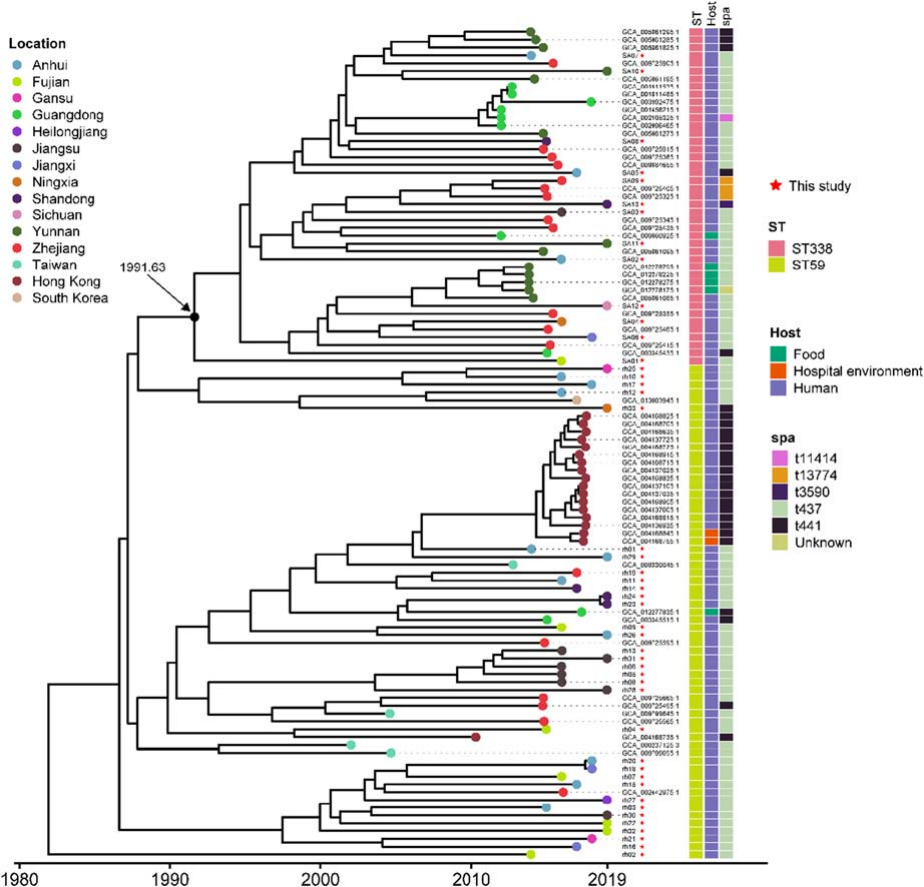
Phylogeny of ST338 isolates. We aligned 43 ST338 genome sequences (13 from our study, 30 from that by NCBI database) and 63 ST59 sequence (33 from our database and 30 from NCBI). Isolate characteristics are shown on the right, including the location, host and Spa type.

We constructed a phylogenetic tree of the 13 ST338 MRSA SCC*mec* Vb isolates and contemporaneous ST338 and ST59 MRSA SCC*mec* Vb isolates from China and adjacent geographic regions in order to estimate the time when the ST338 strain evolved from ST59. We identified 4150 SNPs in the alignment of 43 ST338 genome sequences (13 from this study and 30 from the NCBI database) and 63 ST59 sequences (33 from this study and 30 from the NCBI database); these were used to reconstruct the evolutionary history of the ST338 and ST59 lineages based on a Bayesian dating method in BactDating [31]. The results showed that ST338 isolates probably evolved from ST59 isolates; the median time of emergence of the most recent common ancestor of all ST338 isolates was around 1991.63 (95% highest posterior density [HPD], 1984.39–2002.45) (Figure 2).

We next calculated paired SNP distances between all ST338 SCC*mec* Vb MRSA isolates in this study and ST59 MRSA-SCC*mec* Vb isolates from China and other ST338 MRSA SCC*mec* Vb isolates in the NCBI database. The SNP difference between ST59 and ST338 ranged from 84 to 202 (median=152 and average=151) (Supplemental Figure S1). Notably, in addition to the SNP in the housekeeping gene *gmk*, there were 5 unique nonsynonymous SNPs that differentiated ST338 from the related ST59 clone; these were in genes related to methicillin resistance (*fmtA*) and metabolism of coenzymes (*pdxt*), lipids (*uppS*), and carbohydrates (*odhA*), and 1 SNP encoded a hypothetical protein (Table S2).

### Virulence genes and pathogenicity islands in ST338

**Figure 3.**
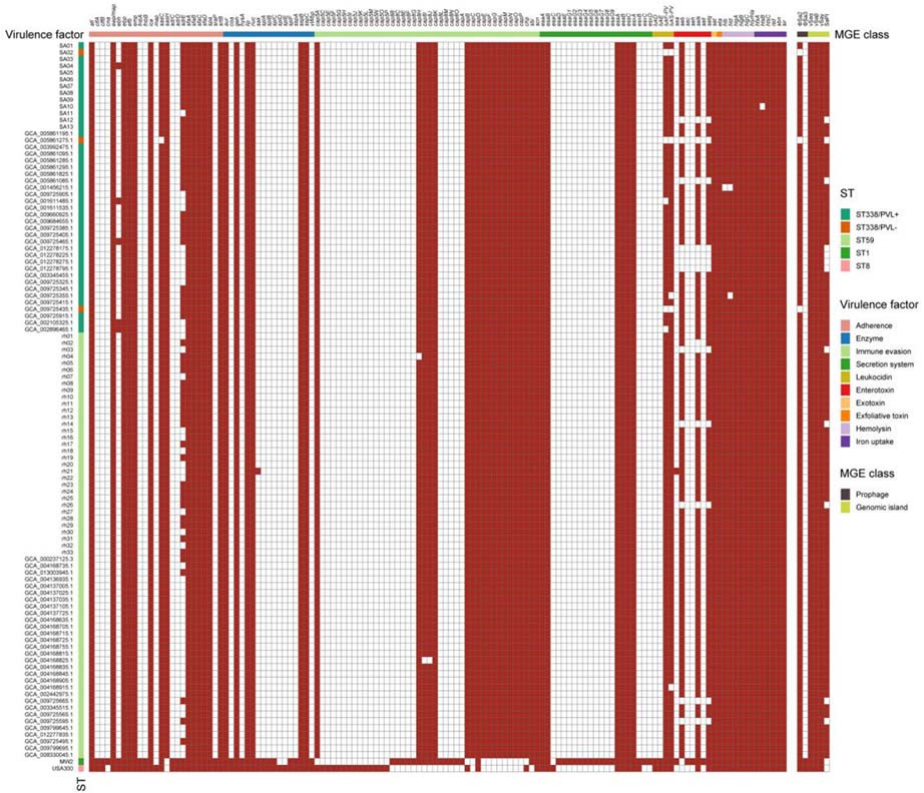
Comparison of virulence factors between ST338, ST59 isolates, MW2 and USA300 strains. Red and white blocks represent the presence and absence of genes, respectively. The horizontal coloured bar represents (from left to right) genes associated with adhesion, serine protease, immune evasion, secretion system, enterotoxin, exfoliative toxin, leukocidin, toxic shock syndrome toxin, exotoxin, and pathogenicity islands.

Virulence genes in the 13 ST338 isolates, rh10 (China mainland ST59-SCC*mec* Vb clone), M013 (Taiwan ST59-SCC*mec* Vb clone), MW2, and USA300 are shown in Figure 3. In general, ST338 isolates shared the same virulence factors and genomic islands that were similar to those of M013 and rh10 but differed from those of MW2 and USA300. Unlike the latter 2 clones, all ST338 strains were negative for the *clfA*, *clfB*, *eap*, *cna*, *sdrC*, and *sdrD* genes encoding adhesion factors, suggesting a weaker adhesion capacity. ST338 isolates also had fewer exotoxin and enterotoxin genes.

**Figure 4.**
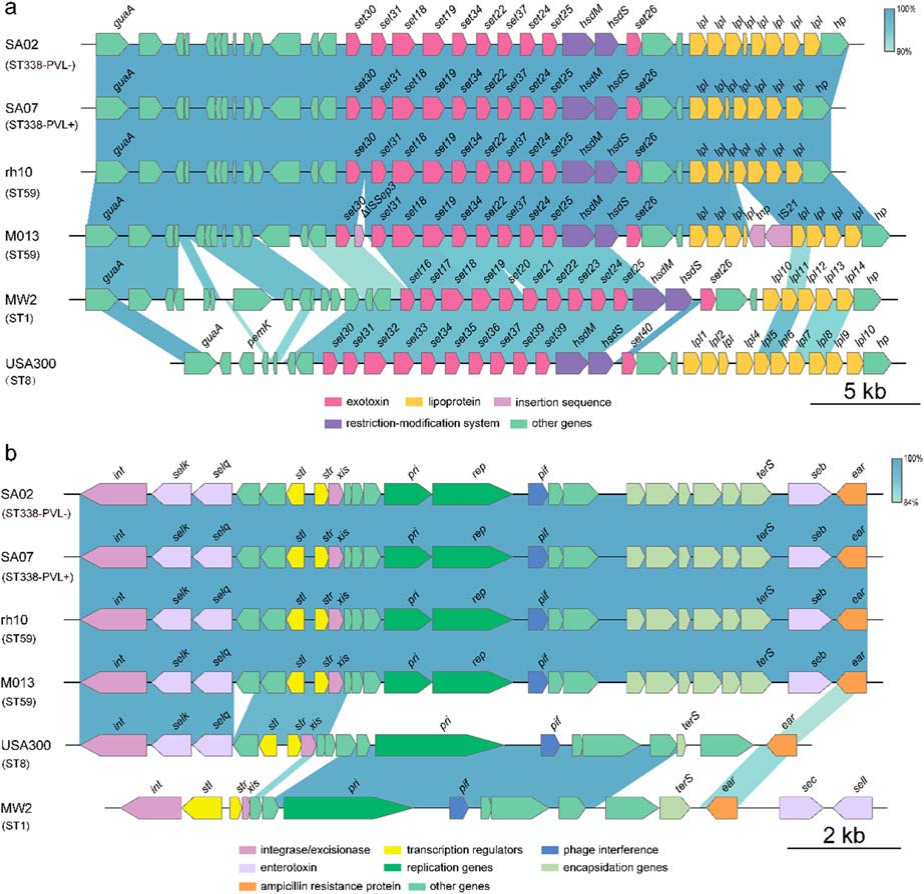
Comparison of the vSaα and SaPI genomic islands between ST338 isolates (ST338-PVL+ and ST338-PVL-), M013 and rh10 (ST59), MW2 and USA300 strains. a, b. Comparison of vSaα (a) and SaPI (b) structure between ST338, rh10, M013, MW2 and USA300. Arrows and arrowheads represent open reading frames (ORFs) and their direction of transcription.

All ST338 isolates harboured the vSaα, vSaβ, and vSaγ genomic islands. Unlike MW2 and USA300, none of the ST338 isolates had νSa3. vSaα encodes a set of putative staphylococcal exotoxin (*set*) genes, a lipoprotein gene (*lpl*), and a restriction/modification system (hsdM/hsdS). However, the *set16*, *set17*, *set20*, *set21*, and *set23* genes present in the vSaα genomic island of MW2 were lacking in ST338 [Figure 4(a)]. νSaβ encodes virulence factors such as serine proteases (*splA–splF*), enterotoxins, and the bicomponent toxin leukocidin LukDE; however, *splA–splF* and LukDE genes were absent in ST338. vSa3 contains *sak* (staphylokinase), *sea* (staphylococcal enterotoxin), *sep* (staphylococcal enterotoxin), and 2 new allelic forms of enterotoxin genes – ie, *sel2* and *sec4*; none of the ST338 isolates harboured *sak* or *sea* (Figure 3). The vSaγ genomic island encoding *set*, *hla* (α-toxin), extracellular fibrinogen-binding protein (*efb*), and *psmβ* (phenol-soluble modulin) is found in most *S. aureus* strains; α-toxin and psmβ are the major virulence factors of *S. aureus*.

SaPIs are mobile genetic elements important for *S. aureus* evolution and virulence. All ST338 isolates harboured SaPI3, which contains *seb*, *selk*, and *selq*; in contrast, MW2 harbours *sea*, *sec*, and *seh* genes but lacks *seb* [Figure 3(b)]. The ΦSa2 prophage – which carries lukF-PV and lukS-PV (PVL), the pore-forming cytotoxins associated with SSTIs and severe pneumonia caused by CA-MRSA [37, 38] – was found in all but one ST338 isolate (SA02). PVL was not detected in SA02, suggesting that the high virulence of ST338 may be independent of PVL status.

The ΦSa3 prophage is not always occur in most of *S. aureus*, such as HA-MRSA strain COL (Gene bank number: CP000046.1). ΦSa3 carries *chp* (chemotaxis inhibitory protein), *scn* (complement inhibitor), *sak* (staphylokinase), and several enterotoxin genes (*sea*, *seg2*, *sek2*, and *sep*). In contrast to MW2, ST338 isolates did not contain *sak* or any enterotoxin genes, but harboured *chp* and *scn* (Figure 3). Thus, the immune evasion cluster type C (ie, *sak*−) is present in νSaβ rather than in ΦSa3 in ST338 clones.

### ST338 strains are less efficient in nasal colonization than MW2

**Figure 5.**
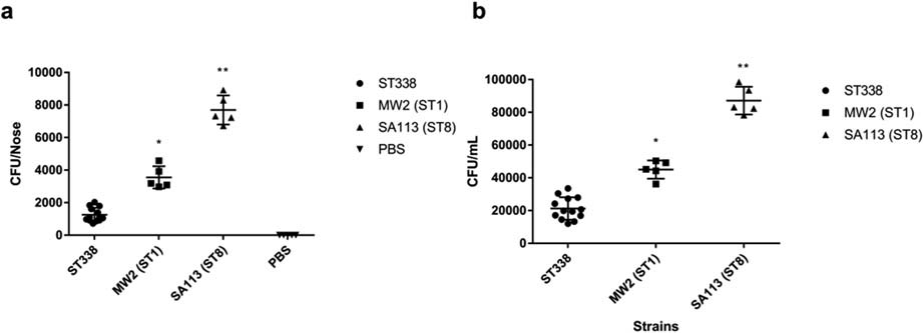
Nasal colonization and cell adhesion capacities of ST338 isolates. (a). Nasal colonization capacity of ST338 isolates compared to MW2 and SA113 strains in mice. Data for the thirteen ST338 isolates in five mice were averaged. (b). Adhesive capacity of ST338, MW2, and SA113 in A549 human alveolar epithelial cells. *P<0.05, **P<0.01.

*S. aureus* mainly colonizes human nasal mucosa. The heatmap of virulence factors suggested that ST338 isolates may have weaker capacity for adhesion than MW2. We evaluated the nasal colonization capacity of the isolates in BALB/c mice with SA113 serving as a positive control, and confirmed that ST338 strains were less efficient in nasal colonization [*P*<0.01; Figure 5(a)]; moreover, they had a lower capacity for epithelial cell adhesion than MW2 [*P*<0.05; Figure 5(b)]. These results combined with the comparative analysis of virulence factors in ST338 and MW2 indicate that ST338 isolates are less efficient at host colonization than other CA-MRSA strains.

### Virulence of ST338

**Figure 6.**
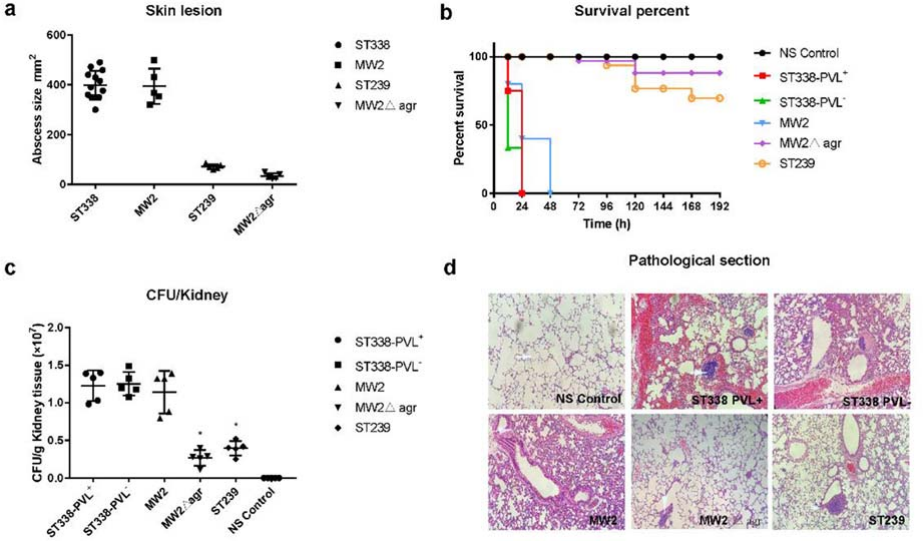
The virulence potential of ST338 isolates *in vivo.* (a). Comparison of invasive capacity among ST338 isolates, MW2, HA-MRSA ST239 strain and the MW2Δ*agr* mutant. Abscess area on day 3 after infection. **P<0.01 (q test). (b). Survival analysis of mice (*n*=10 per isolate) injected with 2×10^9^ CFU or NaCl solution. Survival curves were compared with the log-rank (Mantel–Cox) test. (c). CFUs in kidneys. *P<0.05 (q test). (d). Haematoxylin and eosin staining of lung tissue at 48 h post infection. Inflammatory cell infiltration and tissue damage were greater in ST338 isolates than in HA-MRSA ST239 and the MW2Δ*agr* mutant. A thickened alveolar septum, oedema and congestion, inflammatory cuffs of blood vessels, and leukocyte influx were observed.

According to the case reports, the primary disease caused by ST338 in 9/13 cases was SSTI. To determine the virulence potential of ST338 *in vivo*, we used a mouse skin infection model. The MW2Δ*agr* mutant strain served as a negative control and the low-virulence HA-MRSA ST239 strain was used for comparison. The size of skin abscesses was significantly larger in mice infected with ST338 isolates than in mice infected with MW2Δ*agr* (*p*<0.05), but was comparable to that elicited by MW2 [Figure 6(a)]. This indicates that the ST338-SCC*mec* Vb clone has strong potential to cause invasive skin infections.

We also used a bacteraemia model to investigate the *in vivo* pathogenicity of ST338 isolates [Figure 6(b) and Supplemental Figure S2a]. Mice infected with the PVL+ (SA01) and PVL− (SA02) ST338 strains all died with 24 h (n=10), while those infected with MW2 died within 48 h [Figure 6(b)]. In all cases, infection with the MW2 and ST338 strains caused significantly greater mortality compared to the HA-MRSA strain ST239 and MW2Δ*agr* mutant. The lungs of mice infected with the PVL+ and PVL− ST338 strains showed similar levels of tissue damage to those infected with ST239 and MW2Δ*agr* [Figure 6(d)]. Additionally, histopathologic examination of lung tissue samples revealed greater infiltration of inflammatory cells and tissue damage in the lungs of ST338-infected as compared to MW2-infected mice [Figure 6(d)]. Mice infected with ST338 also showed greater inflammatory cell infiltration in the lungs than those infected with MW2, ST239, or MW2Δ*agr*, with focal alveolar expansion and a thickened alveolar septum that was accompanied by higher CFU counts in the kidneys [Figure 6(c)]. These findings demonstrate the high virulence of the ST338 isolates. It is worth noting that the virulence potential of the PVL− strain (SA02) was similar to that of other ST338 PVL+ strains, suggesting that the high virulence of ST338 strains mainly does not depend on their PVL status.

### Core-genomic virulence genes are highly expressed in CA-MRSA ST338 isolates

**Figure 7.**
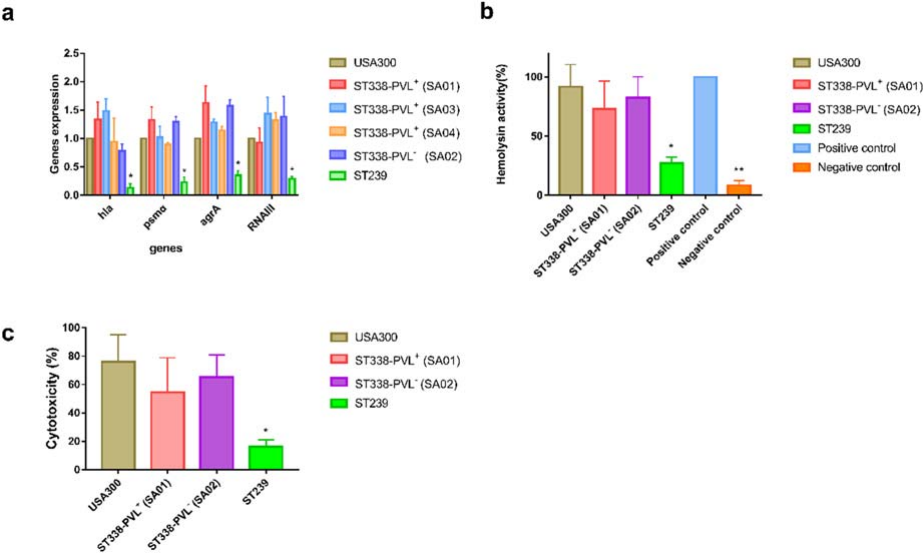
Expression of virulence toxins and cytotoxicity of ST338 isolates. a. Expression of *hla*, *psmα*, *agrA*, and *RNAIII* in ST338 isolates compared to USA300 and ST239 strains. b. α-Toxin activity and production in ST338 isolates compared to USA300 and ST239 strains. c. Cytotoxicity of ST338 in human neutrophils compared to USA300 and ST239 strains. Values represent mean±SD of 3 independent experiments. *P<0.05, **P<0.01.

Given that the *agr*, *hla*, and *psmα* genes contribute to the virulence of CA-MRSA ST59 [22], we investigated whether the high virulence of ST338 strains was due to upregulation of genome-encoded virulence factors. We examined the expression of genes encoding the toxins most frequently linked to CA-MRSA virulence including α-toxin (*hla*), PSMα peptides (*psmα*), *agrA*, and *RNAIII* in three PVL+ and one PVL− ST338 strain. All 4 genes were expressed at levels comparable to those in USA300 [*P*>0.05; Figure 7(a)], although they were more highly expressed in ST338 isolates than in the ST239 strain. We also evaluated α-toxin production and cytotoxicity in ST338 and found that the isolates had significantly higher haemolytic capacity and cytotoxicity than HA-MRSA strains, but were comparable to USA300 in these aspects [Figure 7(b,c)]. Thus, elevated expression of core virulence factors may be responsible for the high virulence of ST338 isolates.

## Discussion

The continuing increase of CA-MRSA infection rates worldwide is a major public health challenge [39]. USA300 is the epidemic CA-MRSA clone in the US [34], whereas ST59 is the most widespread clone in Asia [22]. As a single-locus variant of ST59, ST338 has not yet been identified as a global pandemic clone, although it is increasingly detected in the Asia-Pacific region, especially in China [15, 20, 40] where sporadic isolation of ST338-SCC*mec* Vb – mainly from children – has been reported [41–43]. The complete genome of one ST338 strain has been published [44].

In this study, we characterized 13 ST338-SCC*mec* Vb strains isolated from 13 severe bloodstream infection cases. The phylogenetic analysis indicated that the earliest ST338 isolate (SA07) may have acquired novel mutations to adapt to different environments. Furthermore, the phylogenetic reconstruction and time estimation suggested that ST338 evolved from ST59 in 1991. Notably, while SNP differences for isolates from the same clonal complex (CC) usually range from 0 to 3000 SNPs, differences between ST59 and ST338 ranged from 84 to 202 although both belong to CC59, indicating that the CA-MRSA ST338 SCC*mec* Vb isolates are closely related to the ST59 SCC*mec* Vb clone.

All ST338 isolates as well as ST59-SCC*mec* Vb strains harboured similar virulence factors and genomic islands; however, the former lacked the *clfA*, *clfB*, *ebp*, *cna*, *sdrC*, and *sdrD* genes found in MW2 and USA300. ClfA promotes bacterial colonization and adhesion [45]; *clfB*, *sdrC*, and *sdrD* mediate adhesion to human nasal mucosa [46–48]; and Cna is involved in tissue colonization [49]. Experiments in mice and human alveolar epithelial cells showed that ST338 had a lower nasal colonization capacity than MW2, which is consistent with its lack of adhesion factor genes (*clfA*, *clfB*, *ebp*, *sdrC*, and *sdrD*).

The precise determinants of the high virulence potential of CA-MRSA strains are unclear [12, 50]. It has been suggested that CA-MRSA but not HA-MRSA strains acquire the PVL-carrying mobile genetic element [51], and that the expression of core genome-encoded toxin genes is upregulated in CA-MRSA strains [52]. In this study, we analysed the virulence of ST338 isolates, of which twelve were PVL+ and one was PVL−, and found that they had comparable pathogenicity to MW2. We established mouse infection models to evaluate the invasive capacity of ST338 isolates; the results showed that ST338 strains were slightly more effective in causing SSTI than MW2, although the difference was nonsignificant; and the virulence potential of ST338 strains was comparable to that of MW2. Moreover, the virulence potential of the PVL− strain (SA02) was similar with that of PVL+ strains, implying that PVL status may be unrelated to the high virulence of ST338.

The significance of PVL status for CA-MRSA infection is still unclear. A highly virulent PVL− CA-MRSA strain (ST72) has been reported [53], and the colonization ability of a PVL deletion mutant was found to be comparable to that of the parent strain in an *in vivo* infection model [12]. We examined whether the high virulence potential of ST338 isolates depends on elevated expression of virulence factors using USA300 as a reference strain because of its high levels of related genes [34, 35]. We found that the levels of *hla*, *agrA*, *psmα*, and *RNAIII* in ST338 isolates were comparable to those in the USA300 clone, demonstrating that the high expression of virulence factors in ST338 isolates may be responsible for their virulence, which was also supported by *in vitro* data.

In conclusion, this study provided the first detailed characterization of 13 ST338-SCC*mec* Vb CA-MRSA strains isolated from severe bloodstream infection cases. We demonstrated that the high virulence potential of these strains may be attributable to elevated expression of genome-encoded toxin genes. We also confirmed the emergence of a PVL− CA-MRSA ST338 strain that can cause severe SSTI comparable to PVL+ CA-MRSA strains. Our findings provide insight into the epidemiology of the novel and highly virulent ST338-SCC*mec* Vb clone. As the clinical and experimental data suggest that ST338-SCC*mec* Vb can cause serious and even fatal infections, its transmission should be monitored. However, because we did not investigate the factors contributing to the epidemiologic success of CA-MRSA isolates, it is difficult to predict whether ST338 CA-MRSA has epidemic potential like ST59.

## Supplementary Material

Supplemental Material
